# Pharyngeal Myiasis Caused by Sheep Botfly, *Oestrus ovis* (Diptera: Oestridae) Larva, Tabriz, East Azarbaijan Province, Iran: a Case Report

**Published:** 2017-03-14

**Authors:** Teimour Hazratian, Ali Tagizadeh, Mohammad Chaichi, Madineh Abbasi

**Affiliations:** 1 Departement of Parasitology, School of Medicine, Tabriz University of Medical Sciences, Tabriz, Iran; 2 Departement of Emergency Medicine, School of Medicine, Tabriz University of Medical Sciences, Tabriz, Iran; 3 Departement of Infectious and Tropical Diseases, School of Medicine, Tabriz University of Medical Sciences, Tabriz, Iran

**Keywords:** Myiasis, Pharyngeal myiasis, *Oestrus ovis*, Iran

## Abstract

Myiasis is caused by the larvae of flies infesting animal or human tissues and organs. This report aims to present a case of pharyngeal myiasis caused by the larvae of *Oestrus ovis* (Diptera: Oestridae). A 55-yr old drug addict living in the Shahindeje village of Western Azerbaijan Province, northwestern Iran was referred to the Emam Reza Hospital in Tabriz, having a medical history of Chronic Obstructive Pulmonary Disease (COPD) and hospitalized due to respiratory distress, 20 days ago. He was intubated with a mechanical ventilator (MV) because of his respiratory distress condition. There was an evidence of the presence of pulmonary nodules in his lungs following diagnosis, and a CT scan revealed a cavity in his lung. During the nasogastric intubation procedure, a larva was seen emerging from the patient’s mouth by one of the staff of the intensive care unit of the hospital. A laboratory diagnosis was performed in the Entomology Department of the School of Medicine, Tabriz University of Medical Sciences. Interestingly, larvae of *O. ovis* were identified and confirmed following the laboratory proceedings.

## Introduction

Propounded by Hope in 1840, myiais originated from a Greek word known as “Miya” which means fly (Sharma et al. 2008). Myiasis is a term used to describe a medical condition in which the tissues and organs of human or animals are infested by the larvae of flies (Masoodi et al. 2000). These fly larvae periodically obtain food from their hosts’ dead or living tissues, body fluids or directly ingest solid food (Laner and Crosskey 1993), which may result in various clinical manifestations. The sheep nasal botfly, *O. ovis*, is one of the remarkable causative agents of human myiasis (Verstrynge and Foets 2004). The family Oestridae encompasses a wider range of species in which the larvae of all included species are obligate parasites of domestic animals or wildlife.

Unexpected intrusion of human tissues by a number of these parasites gives rise to severe pathological consequences. The development of Oestrinae larvae occur in the nasopharyngeal cavities of a host of mammalian species. First-stage larvae of *O. ovis* are deposited by the females into the nostrils of susceptible domestic mammals. Though uncommon, the larvae are occasionally transferred into the eyes, the mouth, or the external ear of man, especially in people who are in close contact with sheep and goats. Notably, patients complain being hit in the eye by a wandering insect or object. In critical situations, migration of the larvae to the nasal cavities ensues, leading to an abscess formation, pain and headache. In human nasal cavities, the growth of fly larvae into a third stage hardly occurs. When these larvae spread to the throat, swallowing of food becomes challenging, however these symptoms do not last long (Richard and Crosskey 1996).

Classification of myiasis may be accomplished in two wasy, entomological or clinical. In Entomology, myiasis is classified according to the parasitic characteristics of the fly larvae. These include obligatory, facultative and accidental parasitic fly larvae. Clinically, however, myiasis is classified based on the anatomical region in which infestation occurs (Langan et al. 2004). Key examples of the clinical classifications include ophthalmomyiasis (Janbakhsh et al. 1977), Urogenital myiasis (Jdalayer et al. 1978), Orbit myiasis (Khataminia et al. 1999), Pharyngeal myiasis (Karimi and Vahidi 1999), Ear myiasis (Talari et al. 2002), Auricular myiasis (Tirgary et al. 1977), Oral mucosa myiasis (Hakimi and Yazdi 2002) and wound myiasis (Talari et al. 2004). In addition, clinical classification of myiasis may be described as primary or secondary. Rarely seen in humans but frequent in cattle (called bicheiras), primary myiasis results from the invasion of living tissue-feeding (biophagous) fly larvae. Comparatively, secondary myasis develop when necrobiophagous fly larvae invade and feed on dead tissues, commonly seen in humans in which the patients suffer necrotic cavity lesion (Shinohara et al. 2004).

The life cycle of the sheep bot fly begins from the fertilization of eggs in the female which later hatch into 1mm larvae in the female’s body. Subsequently, a few larvae are directly released in a tiny drop of mucus into the nostrils of the animal host, after which the larvae migrate through the mucosa of the nostrils into the nasal sinuses. The maturation of the larvae continues at this stage following their growth and molting into the second larval stage, reaching a full length of 20mm with characteristic dark-striped segments. Upon full maturation, the larva prepares itself for pupation in the ground after it emerges and falls from the nostrils. Temperature plays a crucial role in the determination of the period of larval growth. In warmer weather conditions, larval maturation may be completed within 25–35d, while this may take a much longer time in colder climates up to 10 months. The temperature-dependent, pupal development stage is completed within 3–9 weeks, followed by the emergence of winged adults from the soil and subsequent mating. During the first few weeks (normally 2–4 weeks) after nuptial flight, the adults do not feed but may take up water (Capelle and Kenneth 2012).

Human infestation of the disease has been recognized extensively over the years (Pampiglione et al. 2012). The disease is readily associated with shepherds having close contact with sheep, although a few more cases have been recorded in non-native individuals who harbor the disease and import them into their localities (Masoodi and Hosseini 2004). The disease is medically curable, or corrected with some medication (Gregory et al. 2004).

Hospital-acquired nosocomial myiasis in patients is unusually observed, and oral myiasis occurs at any place, though not very common in developed countries (Reuler et al. 1985). The underlying symptoms of oral myiasis condition entail traumatized face, mouth bleeding, distinctive lesions and debilitation of the palate (Bhatt and Jayakrishnan 2000). Globally, nosocomial myiasis are evident in intensive care units, however, only a few cases are documented due to underreporting of the disease (Joo and Kim 2001). A study by Najjari et al. and Yaghoobi et al indicated the presence of several cases of myiasis in ICU patients in Iran (Najjari et al. 2014), (Yaghoobi et al. 2005). There have been a recent report projecting the rate of nosocomial myiasis infections as 4%, and a mortality rate of 1.3% (Alizadeh et al. 2014).

This novel case report highlights a case of pharyngeal myiasis caused by the larva of *O. ovis* for the first time in Emam Reza Hospital, Tabriz, Eastern Azerbaijan, Iran.

## Case report

A 55yr old addicted Iranian man residing in the Shahindeje villages of West Azerbaijan Province, northern Iran and referred to the Emam Reza Hospital of Tabriz with clinical history of Chronic Obstructive Pulmonary Disease (COPD) for 4yr was hospitalized due to respiratory distress, 20 days ago. Urgent treatment was given, including nasogastric intubation with a mechanical ventilator (MV) due to his respiratory distress condition. Pulmonary nodules were observed in his lungs following diagnosis, and a CT scan revealed cavities in his lungs. During the nasogastric intubation procedure, a larva was seen emerging from the patient’s mouth by an ICU staff of the hospital, 30 days after hospitalization. This patient having a clinical history of COPD finally died a few moments later.

Laboratory diagnosis was performed in the Entomology Department of the School of Medicine, Tabriz University of Medical Sciences. Interestingly, larvae of *O. ovis* were identified and confirmed following the laboratory proceedings. Precisely, the larva of *O. ovis* was identified as follows; the first segment (head) carried a highly sclerotized tissue surrounding the opening of the respiratory canal followed by 5 rides and two caudal swellings.

Myiasis of this type has not been previously reported from Tabriz. This case report is the premier report of human pharyngeal myiasis caused by *O. ovis*. The majority of myiasis cases reported in the literature from Iran were nasal myiasis originating from various areas. The agents of these cases were *Chrysomyia bezziana*, *Lucilia sericata* and *Erystalis tenax* (Diptera: Syrphidae), (Youssefi et al. 2012). *Lucilia sericata* (Diptera: Calliphoridae) and *Ch. bezziana* (Diptera: Calliphoridae) have been identified as causative agents of wound myiasis in Iran (Alizadeh et al. 2014). Ophthalmomyiasis is an infestation of human eyes and orbital tissues with fly larvae belonging to the botfly species of *O. ovis*, and auricular myiasis is caused by *Ch. bezziana* and *Lu. sericata* (Akbarzadeh 2012). However, oral myiasis arises from the infestation of obligatory parasitic fly larvae of *O. ovis* (Diptera: Oestridae) and *Wohlfahrtia magnifica* (Diptera: Sarcophagidae) (Alizadaeh et al. 2014). Ingestion of whole flies leads to intestinal myiasis or accidental myiasis in human. In Iran, two fly species of *Sarcophaga haemorrhoidalis* and *Er. tenax* are the main causative agents of intestinal myiasis (Khalili et al. 2007, Mandell et al. 2010). Another type of myiasis, the urogenital myiasis, rarely occurs in Iran, and two cases of this type have been reported so far in the country in which the larvae of *Ch. bezzizna* and *Wo. Magnifica* were detected (Salimi et al. 2010). All nosocomial myiasis infections recorded in Iran are of the nasal type caused by facultative myiasis agents, *Lu. sericata* (Diptera: Sarcophagidae) (Mowlavi et al. 2011).

This current study illustrates several interesting facts. First, the dwelling of the patient in poor hygienic conditions of a rural setting was a predisposition factor for larval infestation. Second, lack of awareness also contributed to this condition in the patient.

**Fig. 1.**
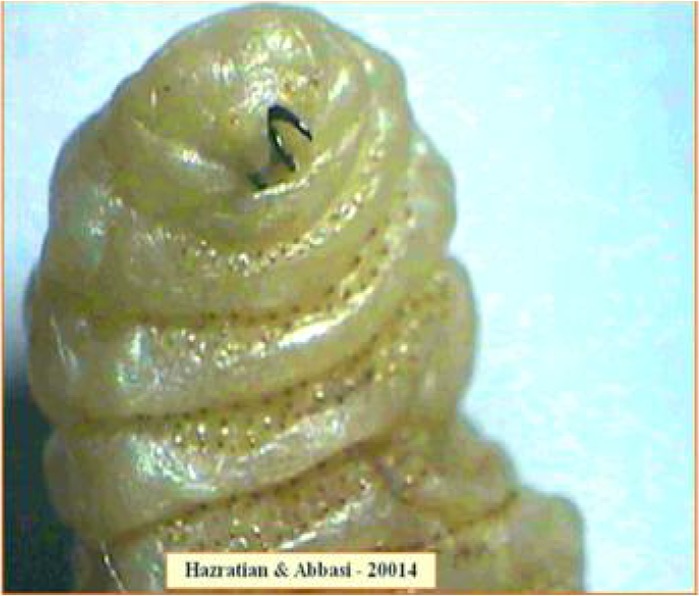
The pair of sharply curved mouth hooks and stout spines of the larva of *Oestrus ovis* according to the laboratory diagnosis

**Fig. 2.**
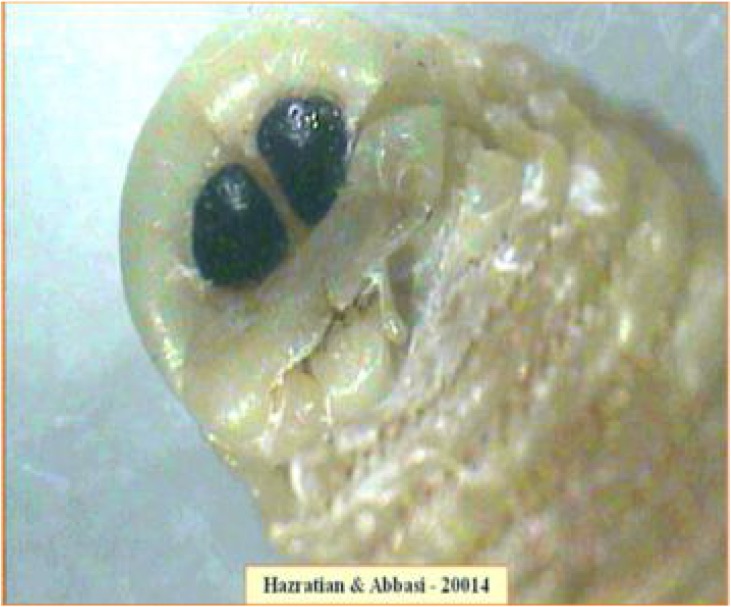
Posterior spiracles in larvae of *Oestrus ovis* without 3 distinct slits and not divided into several plates

## Conclusion

This is by our knowledge the first report of an imported case of pharyngeal myiasis caused by *O. ovis* in northwestern Iran. Myiasis of this type has not been previously reported from Tabriz, and this case of human pharyngeal myiasis caused by *O. ovis* is primarily reported for the first time in Iran. The larva belonged to the species of *O. ovis* according to the morphological characters of larva in which the first segment was characterized by a strongly sclerotized tissue surrounding the outlet of the respiratory canal, completely followed by 5 rides and two caudal swelling.

## References

[B1] Akbarzadeh K ( 2012) Estimation of geographical distribution, biodiversity and species richness of myiasis inducing flies in Fars Province. Tehran University of Medical Sciences, School of Public Health, Tehran, Iran.

[B2] Alizadeh M Mowlavi Gh Kargar F Nateghpour M Akbarzadeh K Hajenorouzali M ( 2014) A review of myiasis in Iran and a new nosocomial case from Tehran Iran. J Arthropod Borne Dis. 8( 2): 124– 131. 26114125 PMC4478423

[B3] Amendt J Lee Goff M Campobasso Cp Grassberger M ( 2010) Current concepts in forensic Entomology. Springer, Dordrecht Chapt 16. p. 355.

[B4] Bhatt AP Jayakrishnan A ( 2000) Oral myiasis: a case report. Int J Paediatr Dent. 10( 1): 67– 70. 11310129 10.1046/j.1365-263x.2000.00162.x

[B5] Capelle Kenneth J ( 1966) The Occurrence of *Oestrus ovis* L. in the Bighorn Sheep from Wyoming and Montana. J Parasitol. 52( 3): 618– 621. 5942536

[B6] Gregory AR Schatz S Lambaugh H ( 2004) Ophthalmomyiasis caused by the sheep bot fly, *Oestrus ovis*, in northern Iraq. Optom Vis Sci. 81( 8): 586– 90. 15300116 10.1097/01.opx.0000141793.10845.64

[B7] Hakimi R Yazdi I ( 2002) Oral mucosa myiasis caused by *Oestrus ovis*. Arch Iran Med. 5( 3): 194– 196.

[B8] Jdalayer T Maleki M Moghtader M ( 1978) Human urogenital myiasis cause by *Chrysomyia bezziana*. Iran J Public Health. 7( 3): 116– 118.

[B9] Janbakhsh B Pirous MS Tirgari S Aghamohamadi A ( 1977) A case of ophthalmomyiasis in man by *Oestrus ovis* in Tehran (Diptera: Oestridae). Acta Med Iran. 20: 19– 26. 614770

[B10] Joo CY Kim JB ( 2001) Nosocomial submandibular infections with dipterous fly larvae. Korean J Parasitol. 39( 3): 255– 260. 11590916 10.3347/kjp.2001.39.3.255 PMC2721075

[B11] Karimi GH Vahidi MR ( 1999) A case report of pharyngeal myiasis. J SSUMS. 1( 7): 91– 94.

[B12] Khalili B Ebrahimi M Khoobdel M ( 2007) A case of intestinal myiasis due to *Sarcophaga hemorrhoidalis* from Chahar Mahal and Bakhtiari Province. J Shahrkord Univ Med Sci. 9( 2): 85– 88.

[B13] Khataminia GH Elyasi P ( 1999) Advanced orbital myiasis: Acase report in Sina Hospital, Ahwaz. SJEB of Iran. 1( 5): 59– 61.

[B14] Lane RP Crosskey RW ( 1996) House -fly, blow-flies, and their allies (Calyptrate Diptera). In: Lane RP Crosskey RW (1996) Medical insects and arachnids. London Chapman and Hall Text Book, pp. 429– 469.

[B15] Langan SM Dervan P Loughlins O ( 2004) A moving scalp nodule in a returning travel. Br J Dermatol. 151( 6): 1270. 15606526 10.1111/j.1365-2133.2004.06354.x

[B16] Mandell GL Bnnett JE Dolin R ( 2010) Principles and Practice of Infectious Diseases. Vol. 2 Elsevier Churchill Livingston, Philadelphia.

[B17] Masoodi M Hosseini K ( 2004) External ophthalmomyiasis caused by sheep botfly (*Oestrus ovis*) larva. Arch Iran Med. 7 ( 2): 136– 139.

[B18] Mowlavi Gh Nateghpour M Teimoori S Amin A Noohi F Kargar F ( 2011) Fatal nosocomial myiasis caused by *Lucilia sericata*. J Hosp Infect. 78: 338– 339. 21684632 10.1016/j.jhin.2011.04.005

[B19] Najjari M Shafiei R Fakoorziba MR ( 2014) Nosocomial myiasis with Lucilia sericata (Diptera: Calliphoridae) in an ICU patient in Mashhad, Northeastern of Iran. Arch Iran Med. 17( 7): 523– 525. 24979568

[B20] Pampiglione S Gianetto S Virga A ( 1997) Persistence of human myiasis by oestrus ovis L. among shepherds of the Etnean area (Sicily) for over 150 years, Dipartimento di Sanitá Publica Veterinaria e Patologia Animale, Univ. di Bologna, Italy. 39( 4): 415– 418. 9802104

[B21] Pinto C Vairo K de Mello-Patiu CA de Cavalho CJB ( 2011) Pictorial identification key for species of Sarcophagidae (Diptera) of potential forensic importance in southern Brazil. Rev Bras Entomol. 55( 3): 333– 347.

[B22] Reuler JB Girard DE Cooney TG ( 1985) Current concepts. Wernicke’s encephalopathy. N Engl J Med. 312( 16): 1035– 1039. 3885034 10.1056/NEJM198504183121606

[B23] Salimi M Goodarzi D Karimfar MH Edalat H ( 2010) Human urogenital myiasis caused by *Lucilia sericata* (Diptera: Calliphoridae) and *Wohlfahrtia magnifica* (Diptera: Sarcophagidae) in Markazi Province of Iran. Iran J Arthropod-Borne Dis. 4( 1): 72– 76. 22808392 PMC3385545

[B24] Sharma J Panchakarappa G Acharya R ( 2008) Primary oral myiasis. Med Oral Patol Oral Cir Bucal. 13( 11): E714– E716. 18978712

[B25] Shinohara EH Martini MZ Oliveira Neto HG Takahashi A ( 2004) Oral myiasis treated with Ivermectin. Braz Dent J. 15( 1): 79– 81. 15322651 10.1590/s0103-64402004000100015

[B26] Talari Sa Yeganeh Moghadam A Dehghani R ( 2002) *Chryzomya bezziana* infestation. Arch Irn Med. 5( 1): 56– 58.

[B27] Tirgari S Khalkhali K Aghamohamadi A ( 1977) First case of auricular myiasis due to *Sarcophaga haemorrhoidalis* (Diptera: Sarcophagidae). Ent Mon Mag. 112: 255– 256.

[B28] Verstrynge K Foets B ( 2004) External ophthalmomyiasis: a case report. Bull Soc Belge Ophtalmol. (294): 67– 71. 15682921

[B29] Yaghoobi R Tirgari S Sina N ( 2005) Human auricular myiasis caused by *Lucilia sericata*: clinical and parasitological considerations. Acta Med Iran. 43: 155– 157.

[B30] Youssefi MR Rahimi Mt Mahaba Z ( 2012) Occurrence of nasal nosocomial myiasis by *Lucilia sericata* (Diptera: Calliphoridae) in north of Iran. Iran J Parasitol. 7( 1): 104– 108. PMC3488829 23133480

